# Polyphosphate Polymerase—A Key Enzyme for the Phosphorus Economy of the Microalgal Cell and the Sustainable Usage of This Nutrient

**DOI:** 10.3390/plants14193061

**Published:** 2025-10-03

**Authors:** Alexei Solovchenko

**Affiliations:** Faculty of Biology, Lomonosov Moscow State University, Leninskie Gory 1/12, Moscow 119234, Russia; solovchenkoae@my.msu.ru

**Keywords:** microalgae, polyphosphate, luxury uptake, phosphorus bioremoval

## Abstract

Phosphorus is a key macronutrient central to the processes of energy and information storage and exchange in the cell. Single-celled photosynthetic organisms, including microalgae, accumulate intracellular reserves of phosphorus (mostly in the form of polyphosphate) essential for the maintenance of cell homeostasis during fluctuations of external phosphorus availability. The polyphosphate reserves in microalgal cells are formed by polyphosphate polymerases—a ubiquitous enzyme family represented mainly by prokaryotic (PPK-type, typical of prokaryotes, e.g., cyanobacteria) and VTC-type polyphosphate polymerases harbored by eukaryotic microalgae, although certain species possess both PPK and VTC types of the enzyme. This enzyme is important for the environmental fitness of microalgae dwelling in diverse habitats, as well as for the efficiency of microalgae-based systems for the biocapture of phosphate from waste streams and for upcycling this valuable nutrient to agricultural ecosystems via biofertilizer from microalgal biomass. This review summarizes the recent progress in the field of structure, regulation, and functioning of VTC in microalgae. In conclusion, biotechnological implications and perspectives of VTC as a target of microalgal cell engineering and bioprocess design for improved phosphate bioremoval efficiency and culture robustness are considered.

## 1. Introduction

Phosphorus (P) is a key macronutrient central to the processes of energy and information storage and exchange in the cell [1,2,3]. The most bioavailable form of P is inorganic phosphate, P*_i_*, which is directly involved in many metabolic reactions [4,5,6]. Both shortage and excess of P*_i_* are deteriorative for normal functioning of the cell. Therefore, the maintenance of P*_i_* homeostasis is essential for the normal operation of biological systems, including photosynthetic organisms [7,8,9]. This goal is accomplished through the concerted operation of P*_i_* uptake via the P*_i_* transporter system and turnover of P reserves in the cells to match its metabolic demand vs. availability of P [10]. As the balancing of supply and demand, stockpiles, and border controls constitute core concepts of economics, P uptake, storage, and turnover of its reserves in the cell can be collectively termed “P economy of the cell”, which is implemented via a sophisticated network of precisely orchestrated mechanisms [8,11]. The bulk of P reserves in single-celled photosynthetic organisms including eukaryotic microalgae and cyanobacteria are represented by inorganic polyphosphate (polyP)—a ubiquitously present, ancient, multifunctional molecule [1,2,12,13,14]. To date, the capability of synthesizing and accumulating polyP was confirmed in more than 100 microalgal species [15]. These species dwell in diverse habitats, including terrestrial (soil), marine, and freshwater environments featuring a broad range of environmental conditions from arctic to hot acidic springs [15]. Such a broad representation indicates that this ability is not of limited significance, e.g., for acclimation to a specific niche, but of fundamental importance.

Experimental evidence accumulated to date supports the importance of the enzymatic machinery implementing turnover of polyP for the maintenance of cell P homeostasis (or, in frame of the analogy drawn above, for the maintenance of a viable P economy of the cell) [1,16]. In addition to this, polyP fulfills a plethora of other functions in different organisms from bacteria to human. Impaired polyP biosynthesis is associated with impaired mitochondrial metabolism, failure of cell apoptosis, and bone mineralization; it also promotes procoagulant and proinflammatory responses and disturbs mTOR signaling (signaling relayed by *mammalian target of rapamycin*-type kinases) [2,17,18,19,20,21,22]. As an efficient ion chelator, polyP modulates intracellular monovalent and divalent cation availability, serves the function of molecular chaperone preventing protein aggregation under stress [22], binds to proteins and regulates their functions, and activates ribosomal protein degradation in response to nitrogen starvation [8,23,24,25]. The latter mechanism is particularly important for the induction of valuable compound biosynthesis in microalgae by nitrogen starvation [26].

In microalgae, polyP is synthesized by polyphosphate polymerase. This enzyme family includes eukaryote-type polyP polymerase (commonly termed VTC since it was first described as vacuolar transport chaperone proteins [27]) and prokaryote-type enzymes, PPK [28]. Certain eukaryotic microalgae harbor both types of enzymes (see below and [29]). Polyphosphate polymerases are conserved throughout microalgae and other major groups of living organisms [27]. Although polyP was found in all cells studied so far [1,12], eukaryotic polyP polymerases, VTC, remained poorly studied due to their complexity, but recent breakthroughs have considerably deepened our understanding of their structure and function [30,31].

The VTC enzyme and VTC-dependent biosynthesis of polyP gained increasing attention from researchers during the last decade (see [15,30,32,33] and references therein). It became clear that the functioning of VTC and its regulation is at the core of P flows and partitioning in individual cells, microbial consortia, and larger ecosystems [16]. The VTC-mediated biosynthesis of polyP is of primary importance for the efficiency of microalgae-based biotechnologies, suggested as a promising solution for the sustainable usage of P. Illustrious examples of those include the biocapture of P*_i_* from waste streams and wastewater treatment facilities with microalgal cultures [33,34,35,36] and re-routing it to agroecosystems via “green” biofertilizer derived from the microalgal biomass [37].

Experimental evidence on the functional significance and biotechnological importance of polyP polymerases in microalgae has been rapidly accumulating during the last decade, while the generalizations on these topics mostly lag. In view of this, recently obtained experimental facts about polyP polymerases of microalgae are summarized below. Towards this end, handpicked reports were carefully selected, both experimental papers and recent reviews reflecting, in our view, the most significant breakthroughs in the field of polyP in microalgae. Certain influential studies were included to help in understanding the developments in the field. The review starts with the general outline of polyP polymerase, its subcellular localization, structure, and function (mostly on the example of VTC), continues to the regulation of this enzyme, and concludes with its implications for biotechnology.

## 2. Localization in the Cell

**Figure 1:**
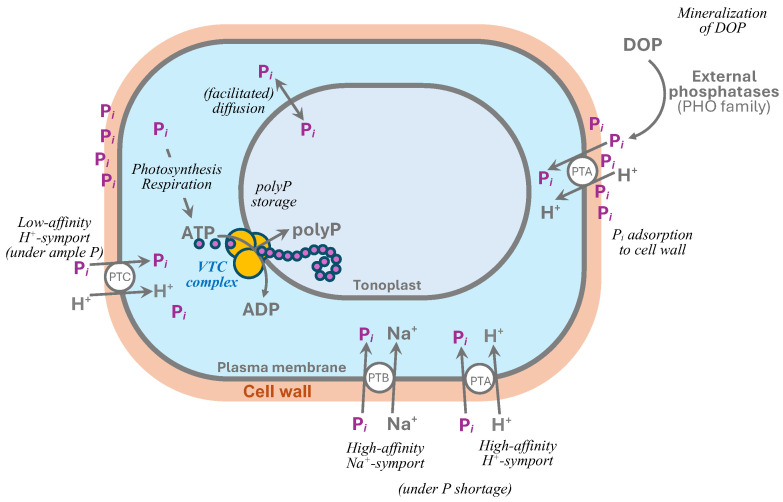
The P*_i_* taken up by the cell in excess of current metabolic demand is converted into polyphosphate with the participation of the VTC enzyme complex. VTC, vacuolar transport chaperone; DOP, dissolved organic phosphorus. Designations: PTA, PTB, PTC,—P*_i_* transporters. Adapted from [37].

Orthophosphate, if taken up in a large amount, e.g., during luxury uptake (uptake above the current metabolic demand) and/or overcompensation response (increased uptake following P shortage) [5,38,39,40,41,42], can potentially displace the equilibria of phosphorylation–dephosphorylation reactions in the cell. Hence, important roles of polyP polymerase in the cell include the conversion of the surplus P*_i_* into polyP and compartmentalization of the latter in a less metabolically active compartment, e.g., vacuole (Figure 1). This function is particularly important, since short-chain polyP synthesized outside the vesicle or vacuolar compartment, preventing direct contact of polyP and cytoplasm, is believed to be toxic for the cell [43,44].

In the eukaryotic cell, polyP polymerase is typically associated with membranes of specialized vacuoles harboring polyP and called acidocalcisomes [45] or acidocalcisome-like vacuoles [46,47,48]. These vacuoles are hallmarked by the presence of polyP in the form of electron-opaque granules, initially described in yeast as “volutin” [49] and colocalized with abundant mono- and divalent cations (e.g., K^+^, Ca^2+^), equilibrating the strong negative charge of polyP [8,11]. At the same time, ultrastructural studies of microalgae frequently reveal the presence of polyP-like granules in numerous other compartments and subcompartments [40,50,51], suggesting the existence of other sites of polyP biosynthesis in the cell. Thus, alternative sites for the initiation of polyP synthesis have been suggested for certain microalgae, e.g., for *Cyanidioschyzon merolae,* where small polyP granules are synthesized in cytosolic vesicles. Later, the small vacuoles merge with larger vacuoles, where big polyP granules are eventually assembled [51]. These peculiarities in polyP biosynthesis were associated with the differences in the genetic determinants of polyP biosynthesis in these organisms (see below).

**Figure 2:**
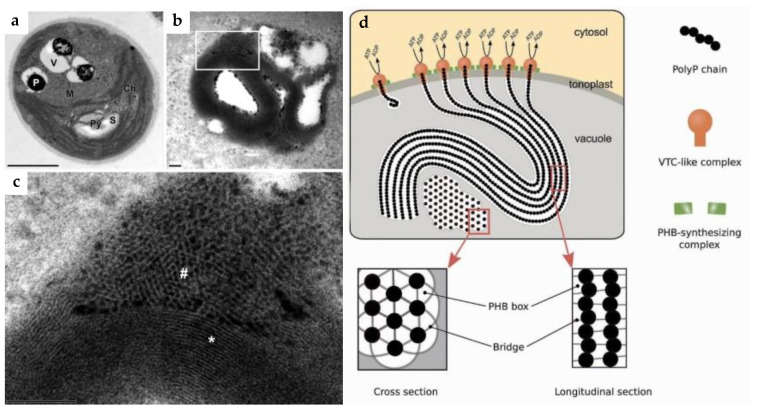
The ultrastructure of *Chlorella vulgaris* CCALA 256 cells with the vacuolar polyphosphate granules from the stationary phase (9 days from re-supplementation of P*_i_* to P-starving cells) culture: (**a**) survey micrograph view of the cells and (**b**) TEM image of the polyP granule in the vacuole and (**c**) its enlarged fragment together with (**d**) a hypothetical schematic representation of the coupled biosynthesis of the polyP chains and their translocation into the vacuole by the tonoplast-bound VTC-like enzyme complexes leading to the formation of the P-rich inclusions. The polyP particle chains are likely enclosed within poly-(R)-3-hydroxybutirate (PHB) and interlinked with bridge-like structures. The “multiwire cable” ultrastructural pattern likely arises from the simultaneous operation of many VTC-like complexes organized as raft(s) floating in the tonoplast. Ch, chloroplast; M, mitochondrion; P, polyP granule; Py, pyrenoid; S, starch grain; V, vacuoles with granules. In c, the longitudinally cut (asterisk) and crosscut (#) regions of the “multiwire cable” chains of the electron-dense particles are shown (see (**d**)). Scale bars, 1 μm (**a**) and 0.1 μm (**b**,**c**). Reprinted from [38] with kind permission from Springer Nature.

Recently, the highly ordered ultrastructure of polyP granules in microalgal vacuoles has been documented [52]. The authors suggested that such a structure can result from the concerted operation of several VTC complexes grouped in the vacuolar membrane into raft-like units. Such VTC arrays simultaneously synthesize several polyP chains wrapped in a sheath of organic substance resembling poly-(R)-3-hydroxybutyrate (PHB), so the whole assembly appeared on TEM images as a “multi-wire electric cable” (Figure 2, see also [38]). The whole picture partially resembled that of the VTC-mediated accumulation of polyP in yeast acidocalcisomes [31] of yeast [44]. As summarized in [38], the sophisticated structure of polyP chains interleaved with PHB was suggested earlier [53] and indirectly confirmed later in *Parachlorella kessleri* NIES-2152 [54].

## 3. Polyphosphate Polymerases in Microalgae

The homologs of the key components of the VTC enzyme complex are conserved in many unicellular eukaryotes [16,29,31] but animals and certain plant lineages (mostly Streptophyta and red algae) lack genes encoding this type of polyP polymerase [55]. The phylogenetic distribution of these genes suggests that the ancestral eukaryote possessed a homolog of the catalytic unit of VTC, VTC4 (see below), but multiple losses have occurred during eukaryotic evolution [16,56].

In vascular plants, the absence of a VTC4 homolog seemingly correlates with the loss of polyP storage capacity: most of their P reserves are stored in the form of P*_i_* in the central vacuole of the cells or as phytic acid [55,56,57,58]. However, this loss does not seem to be complete: the presence of polyP granules in higher plant cells has been reconfirmed recently along with the genes involved in their biosynthesis [56]. Overall, the presence of genes coding for polyP polymerases is considered to be an indication of the capability of polyP biosynthesis. So far, the genes coding for VTC-type polyP kinase were detected in genomes of 48 microalgal species [15], but homology search in genomic databases suggests that many more such discoveries are expected as a result of ongoing genome mining efforts.

**Figure 3:**
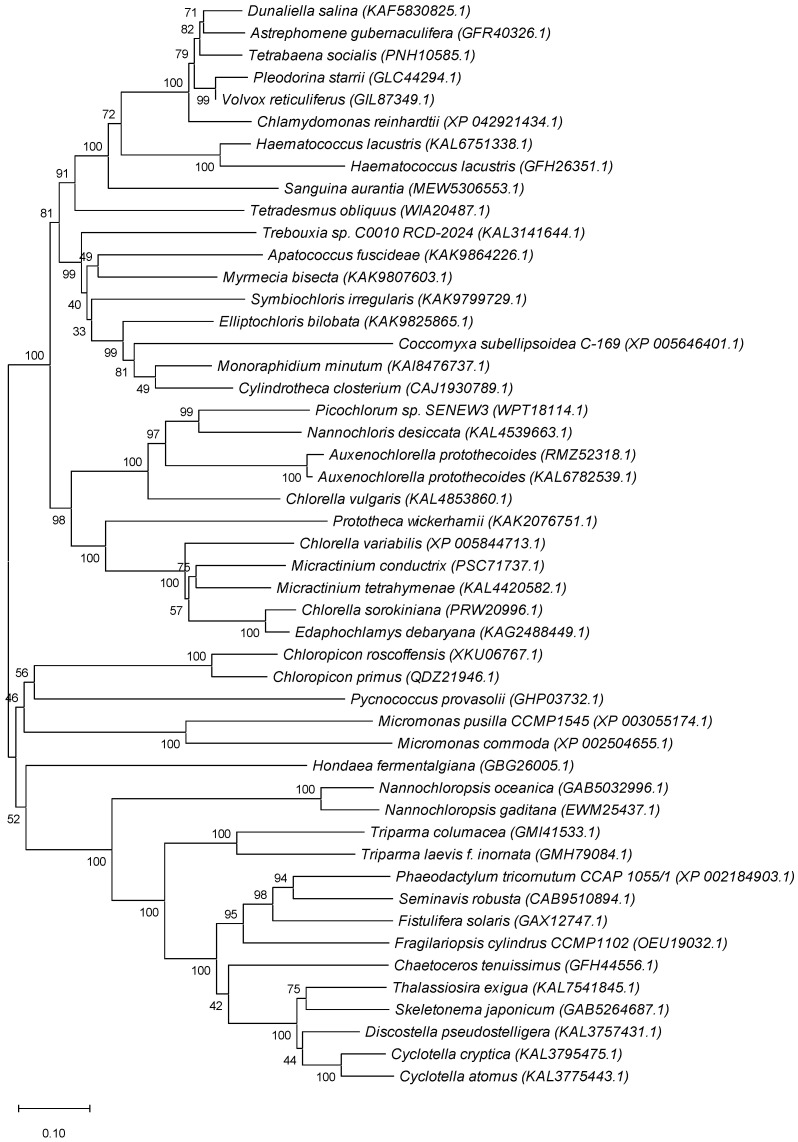
Evolutionary relationships of predicted VTC4 sequences from 49 microalgal sequences from NCBI GenBank (accession numbers are specified in parentheses) homologous to VTC4 of *Chlamydomonas reinhardtii* (XP042921434.1). The evolutionary history was inferred using the neighbor-joining method [59]; bootstrap support values (500 replicates) [60] are shown next to branches. The tree is drawn to scale, with branch lengths in the same units as those of the evolutionary distances used to infer the phylogenetic tree. The evolutionary distances were computed using the Poisson correction method [61]. Evolutionary analyses were completed in MEGA 12 [62].

Current phylogenetic evidence on the putative VTC4-encoding sequences predicted by the homology search with the current NCBI online BLAST tool (see, e.g., Figure 3) and its 3D structure inferred with AlphaFold suggests that the capability of polyP biosynthesis represents a rather ancient ability. Likely, it has evolved with species of the microalgal lineage. Still, the results of the in silico genome mining and other findings made by bioinformatic tools need proper experimental validation.

The importance of this trait for the environmental fitness explains why the functionality of the VTC-type polyP polymerase persisted throughout evolution [15]. Phylogenetic analysis of the predicted VTC4-encoding genomic sequences of microalgae revealed that their phylogenetic relationships largely align with the taxonomic relationships between the species harboring corresponding VTC4 sequences [15]. These similarities and evolutionary relationships suggest that polyP synthesis represents an ancient ability that has evolved as the microalgal lineage has spread out over time. Thus, Cliff et al. [15] showed that VTC4 sequences from *Auxenochlorella protothecoides*, *Micractinium conductrix*, *Volvox reticuliferus*, and *Haematococcus lacustris* appear to be isoforms originating from a single gene for each species. The analysis carried out in the same work demonstrated that species of Chlorellales form two clades, one of which is more closely related to the remaining Chlorophyta sequences than the other clade and differs primarily by the sequences encoding the transmembrane domain. Recent genomic and transcriptomic analyses of the oleaginous microalga *Nannochloropsis oceanica* revealed four genes encoding proteins harboring the SPX domain (named after the first proteins in which it was discovered—SYG1, Pho81, and XPR1), including SPX-VTC (NoSPX2), encoding a protein associated with vacuolar or acidocalcisomal membranes homologous to the VTC4 [63].

A consensus regarding the representation and functionality of polyP polymerases in eukaryotic microalgae is yet to be developed: while certain researchers argue that the VTC is the only known polyP eukaryotic polymerase, there is ground to believe that, at least in some species, VTC is complemented by the homologs of the bacterial-type polyP polymerase [29]. Interestingly, the phylogenetic relationships and representation of PPK1, a highly conserved, bacterial-type polyP polymerase also typical for cyanobacteria [2], scarcely overlaps with the representation of VTC4 homologs. This observation suggests the possibility of differential selection between genes of distinct polyP polymerase types in each lineage [15] (although some chlorophytes possess both homologs [16]).

The results of the genomic studies presented in [64] suggest that polyP kinases of some simple eukaryotic species like microalgae *Ostreococcus tauri*, *O. lucimarinus*, *Porphyra yezoensis*, and *Cyanidioschyzon merolae,* and the moss *Physcomitrium patens,* derived from prokaryotic enzymes, e.g., PPK1 and PPK2 [65]. Most of the PPK homologs harbored by eukaryotic genomes are fused with the genes encoding other enzyme types known as nucleoside diphosphate-linked moiety X (Nudix) hydrolases belonging to the diphosphoinositol polyphosphate phosphohydrolase (DIPP) family, specific to eukaryotes [66]. The genes coding for these enzymes were likely acquired as a result of several individual horizontal transfer events (for more details on the phylogenetic analysis of representation and distribution of PPK homologs in eukaryotes, including microalgae, see [64]). The authors of this work argue that a simpler “prokaryotic version” of polyP polymerase might give an advantage, such as increased tolerance to stresses, including desiccation or nutrient shortage, for the simple eukaryotes that lack more sophisticated adaptations. Acquisition of the “bacterial type” of polyP kinases was hypothetically associated with a boost of marine phytoplankton abilities to sequester P*_i_*, hence accelerating global carbon fixation and transformation of the ancient biosphere.

During recent decades, the functional significance of many proteins harboring SPX domains and involved in the metabolism of P has been revealed. Thus, SPX1-type polypeptides were found to be nuclear repressors of constitutively expressed phosphate starvation response 1 (PHR1); in plants, paralogs of PHR1 (PHL, PHR-like) are the transcription factors of the plant-specific MYB-CC class. Genome-wide analysis of differential gene expression, mostly in the model microalga *Chlamydomonas reinhardtii* [67], revealed many genes and proteins responsible for the acclimation of unicellular green algae to fluctuating P*_i_* availability, collectively referred to as “P*_i_* starvation-inducible” genes or PSI genes. These genes were also found in genomes of marine microalgae, where they encode homologs of the yeast Pho84 and Pho89 high-affinity P*_i_* transporters, the VTC complex, and a broad diversity of enzymes (mostly phosphatases and nucleases), implementing P*_i_* scavenging from surplus RNA and phospholipids [68,69,70].

The most studied VTC4 gene of microalgae is the one in the genome of *Ch. reinhardtii* [13,15,32,68,71,72]. Studies of VTC4 in other species are complicated by incompleteness and/or incorrect truncating of the sequences that were predicted to encode the VTC4 protein and deposited into public databases: they appear to be missing either the N-terminal SPX domain, the catalytic tunnel domain, or the transmembrane domain [15]. Still, homologs of VTC4 were found within > 50 species of microalgae inhabiting marine, freshwater, and terrestrial biotopes. An analysis of protein models generated by Cliff et al. for the predicted VTC4 proteins from four eukaryotic species (*Chlorella vulgaris*, *Desmodesmus* cf. *armatus*, *Gonium pectorale*, and *Chlamydomonas reinhardtii*) demonstrated that the microalgae share the conserved VTC catalytic core and SPX phosphate-sensing domains found in the yeast VTC4 proteins. These microalgae also demonstrated the capability of enhanced polyP accumulation upon the replenishment of P*_i_* to P-starving cultures.

## 4. Structure and Function

The polyP polymerase catalyzes the elongation of the polyP chain with P*_i_* units from ATP; hence, an adequate supply of this substrate is crucial for the biosynthesis of polyP. In eukaryotic cells, the elongation of the PolyP chain takes place along with the translocation of the nascent polyP chain through the vacuole membrane into the vacuole lumen [44]. These mechanistic findings were based on biochemical and genetic evidence and were recently confirmed by structure-guided functional analyses in yeast *Saccharomyces cerevisiae* [30]. The microalgal VTC protein sequences and corresponding yeast protein sequences displayed a high structural homology (38–44% sequence identity with ca. 60% sequence coverage, including the VTC catalytic core), so their predicted structures turned out to be very close, except for their unstructured outer loops [15]. These findings form a basis to believe in the close similarity of the structure and function of these enzymes in yeast and eukaryotic microalgae.

The VTC complex includes the catalytic subunit with PolyP polymerase activity (VTC4) and a set of accessory subunits (Table 1) whose composition depends on the subcellular localization of the complex and the organism [73]. Thus, VTC2 and VTC3 are homologous to VTC4 but are catalytically inactive; VTC1 is homologous to the transmembrane domains of the other subunits [74]. According to current knowledge, microalgae only possess homologues of the VTC1 and VTC4 subunits of *S. cerevisae*, so the structure of the microalgal VTC complex is expected to differ from that of the yeast VTC. The 3D models of the predicted VTC4 proteins generated by Cliff et al. from the four eukaryotic species showed that at least the studied microalgal species share the conserved VTC catalytic core and SPX phosphate-sensing domains initially mapped in the VTC4 proteins of yeast [15]. These findings further confirm the polyP polymerase function of the VTC4 protein in microalgae and support the similarity of VTC4 regulation in microalgae and yeast. Still, the precise details of the assembly of the microalgal VTC complex and the mechanisms in control of this process are not known.

The role of the membrane-integrated VTC complex in the biosynthesis of polyP was initially demonstrated in *S. cerevisiae*. Later, it was proved by reconstitution of an active Vtc1/Vtc3/Vtc4 (a pentamer with the stoichiometric ratio of 3:1:1) complex exhibiting polyP polymerase activity and capable of polyP synthesis activated by inositol (poly)phosphate in vivo [30,44]. In microalgae, the function of VTC4 was initially demonstrated in *Ch. reinhardtii,* albeit no information on its structure was available at that time. Later, the VTC catalytic core and the SPX regulatory domain were shown as conserved among microalgae and yeast, suggesting that VTC4 functions as a polyP polymerase in other microalgae as well. In addition, the affinity of the SPX domain to inositol phosphates was shown via molecular docking calculations [15].

Forward genetics indicated that the components of the polyP polymerase complex, namely VTC1, are involved in acclimation to fluctuations [7] of the availability of other nutrients, such as sulfur (S) or nitrogen (N). Thus, the *ars76* mutant deficient in arylsulfatase is also impaired in acidocalcisome formation and more prone to death under S- or N-deprived conditions compared with wild-type [71].

**Figure 4:**
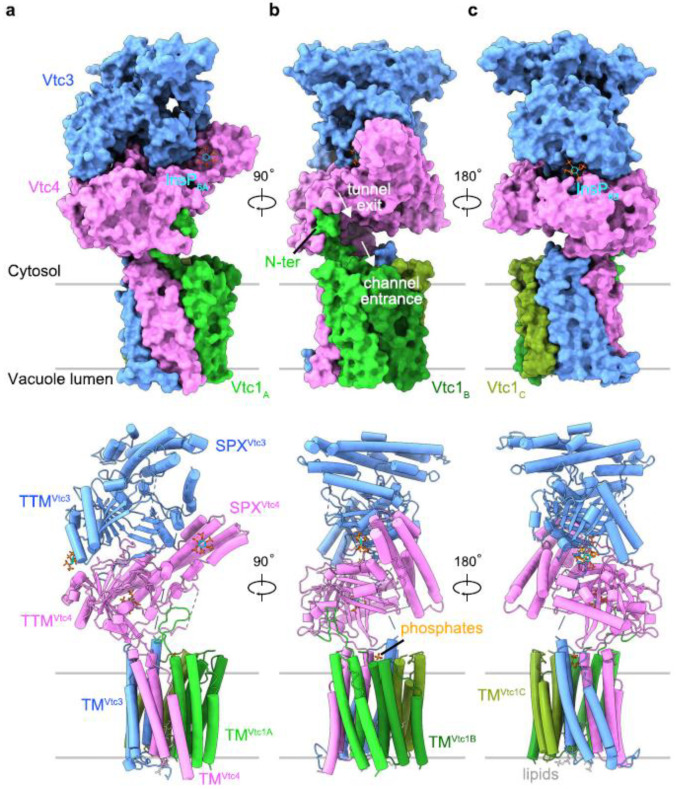
Structure of the InsP_6_-activated VTC complex. (**a**–**c**) Overall structure of Vtc1/Vtc3/Vtc4_R264A/R266A/E426N_ in complex with inositol phosphate, InsP_6_. Surface (**Top**) and cartoon (**Bottom**) representations are displayed in the same perspective. Vtc3 and Vtc4 are colored in blue and pink, respectively. The protomers A, B, and C of Vtc1 are colored separately in gradient green. Molecules of InsP_6_, phosphate and lipid are shown in stick representations. The exit tunnel of catalytic TTM_Vtc4_ and the entrance channel of the transmembrane region are marked with white arrows. Reprinted from [30] under Creative Commons Attribution 4.0 International License (http://creativecommons.org/licenses/by/4.0/: accessed on 1 September 2025).

A recent breakthrough in the structure of the VTC complex was achieved by Guan et al. [30] employing cryo-electron microscopy to resolve the structure of the yeast VTC complex at 3.0 Å resolution (Figure 4). Although the VTC complex was initially predicted to have nine transmembrane helices, a subsequent cryo-EM structural study revealed that the complexes actually possess fifteen transmembrane helices, three helices per subunit. In yeast, 15 transmembrane helices form a novel membrane channel (Figure 4), enabling the transport of a newly synthesized polyP chain into the vacuolar lumen. Notably, the SPX and TTM domains of VTC4 tightly interact (Figure 4a). The exit tunnel of the catalytic TTM domain is positioned at the entrance of the transmembrane channel (Figure 4), the latter is connected to TTM by a well-defined loop interacting with the N-terminus of the Vtc1A protomer at the membrane–proximal region (Figure 4b).

Negatively charged ligands (e.g., P*_i_*, pyrophosphate (PP*_i_*), sulfate, and polyP) can also bind to the TTM domain, enabling the initial binding of PP*_i_* as a primer for the synthesis of polyP [31]. Two P*_i_* ions and two lipid molecules bind to the cytoplasmic and luminal side of the VTC transmembrane channel, respectively (Figure 4b,c).

Bacterial-type polyP polymerase, PPK1, is a dimer forming a catalytic tunnel, in which polyP is elongated by the transfer of P*_i_* from ATP, and the elongating polyP chain exits from the other side [75]. The amino acid sequence of PPK1 consists of highly conserved amino- and carboxy-terminal domains (N and C domains) and a less conserved middle ‘head’ (H) domain; the N and C domains provide the binding site for ATP, and the C domain is responsible for the catalytic function [75]. In addition to the forward reaction of polyP chain elongation, PPK1 catalyzes the reverse reaction (cleavage of P*_i_* from the PolyP chain), as well as nucleoside diphosphate kinase reactions with P*_i_* transferred from PolyP and 5′-tetraphosphate by transferring of PP*_i_* to guanosine diphosphate [76].

## 5. Regulation of Polyphosphate Polymerase Function

Crucial insights into the regulation of VTC functioning were achieved only recently (see, e.g., [30]). In yeast and eukaryote microalgae, polyP synthesis is regulated by inositol pyrophosphate (PP-InsP) nutrient messengers directly sensed by the VTC complex. In eukaryotic microalgae, as in yeast [16] or animals (and humans) [77], the cell P*_i_* level and also its energy status are manifested by signal molecules normally present in small (<5 µM) concentrations. Inositol pyrophosphates, PP-InsP, like 1,5-IP_8,_ can serve as an example. Thus, the SPX domains of VTC3 and/or VTC4 subunits of the VTC complex can bind PP-InsP molecules to the surface collectively formed by basic amino acid residues called the phosphate binding cluster (PBC) and the lysine surface cluster (KSC), which together provide a binding surface for PP-InsPs (for more detail, see [30,31]). In addition to that, the InsP6-activated VTC complex binds molecules of InsP_6_.

Overall, the VTC complex harbors three InsP_6_ molecules: one bound to the basic surface patch of the PP-InsP-sensing SPX domain (Figure 4a), the second bound simultaneously to the tunnel domains of VTC3 and VTC4, likely promoting their association (Figure 4c), and the third InsP_6_ was found in the positively charged tunnel domain of VTC4, in a binding site that is normally occupied by the ATP substrate. Binding PP-InsP results in the orientation of the catalytic polymerase domain at the entrance of the transmembrane channel, activating the enzyme and coupling PolyP synthesis with its translocation through the vacuolar membrane (see Figure 4 in [30]).

Coupling of PP-InsP with the P-level is implemented via two-stage phosphorylation: first of IP_6_ to 5-IP_7_ (by inositol phosphate kinase 1, ITPK1), then 5-IP_7_ to 1,5-IP_8_ (by VIH1/VIH2 or Vip1 homolog kinases, see Figure 4). Replete P*_i_* and replete ATP in the cell boost VIH kinase activity, while P*_i_* shortage reciprocally fosters phosphatase activity of the VIH kinase [78,79]. The net results of this, as summarized in [16], is the oscillation of the energy metabolites in synchrony with derivative signal molecules to adjust and orchestrate metabolism according to P*_i_* supply. The term “INPHORS pathway” was coined for the collective designation of these processes [80].

Interestingly, inositol pyrophosphates assumed the role of central regulators of P*_i_* homeostasis in animal and plant-like *Arabidopsis thaliana* and *Oryza sativa* [80] cells as well. The structural similarity of polyP polymerases in microalgae and yeast supports the involvement of PP-InsPs in the control of polyP biosynthesis and storage in the vacuole in a similar way as was demonstrated in the latter organism [81,82]. The SPX-type receptors were also encountered in plant and human P*_i_* transporters, indicating potential similarities in the mechanisms of their regulation. The SPX sensor domains responsive to P*_i_* in the cell interior were discovered in four plant protein families. They harbor a single SPX domain constituting the entire protein (these proteins were named ‘stand-alone’ SPX polypeptides exemplified by SPX1–SPX4 proteins of *A. thaliana*) or occupying the N-terminal region followed by additional domains. The latter protein type includes the SPX–MFS protein (PHT5/VPT) family, SPX-EXS proteins (PHO1 family), and SPX–RING proteins (NLA family) [83,84].

**Figure 5:**
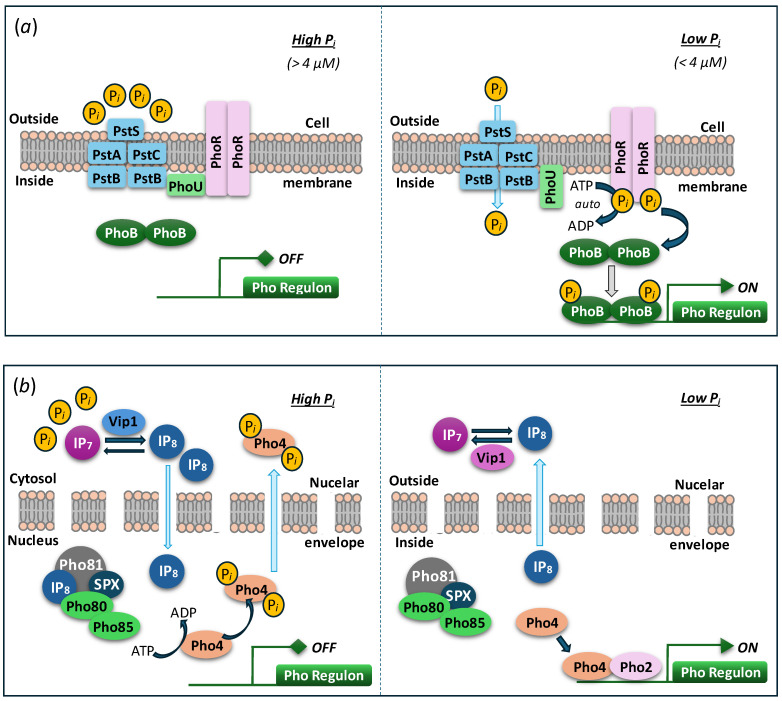

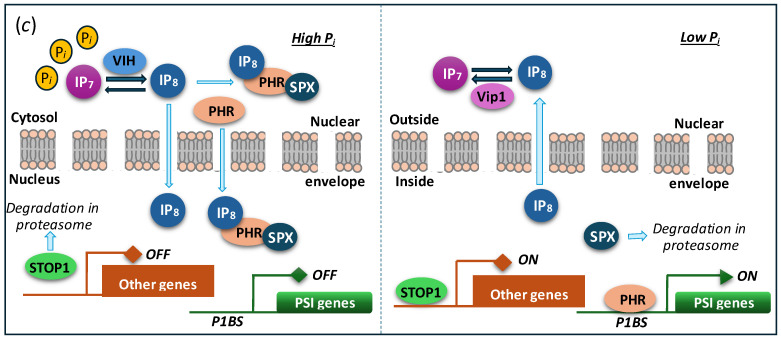
**Minimal models of P*_i_* sensing and core transcriptional response circuits.** (**a**) Pho regulation in bacteria (presumably, including cyanobacteria). The P*_i_*-specific transporter (Pst), composed of four different Pst subunits (blue), functions as a sensor of extracellular P*_i_*. In P*_i_* sufficiency, P*_i_* binds to PstS and inactivates PhoR, a histidine sensor kinase, via PhoU-mediated recruitment to PstB. In P*_i_* limitation, P*_i_* is taken up by Pst, which activates autophosphorylation of PhoR and subsequent phosphorylation and activation of PhoB, a transcriptional response regulator of diverse Pho box-containing promoters (Pho regulon). (**b**) Pho regulation in yeast (*S. cerevisiae*). In P*_i_* sufficiency, Vip1, a bifunctional IP7 kinase/IP8 phosphatase, signals intracellular P*_i_* status via the dynamics of the IP8 level to the cell nucleus. IP8 binds to the SPX domain of Pho81, an inhibitor of the cyclin-dependent protein kinase complex, Pho85 (kinase)–Pho80 (cyclin), which causes partial Pho81 dissociation and thus Pho85–Pho80 kinase activation. Phosphorylation of Pho4, a basic helix–loop–helix transcription factor, facilitates nuclear Pho4 export and Pho gene repression. In P*_i_* limitation, the IP8 level drops (activation of Vip1 IP8 phosphatase), which represses Pho85–Pho80 kinase activity via tighter binding to Pho81. Dephosphorylated, nuclear Pho4 interacts with Pho2, a homeodomain transcription factor, to activate Pho gene expression. (**c**) Regulation of PSI genes in land plants (*A. thaliana*). Like in yeast, the dynamics of IP7/IP8 levels, responding to intracellular Pi and energy status via the bifunctional kinase/phosphatase VIH, control the activation of P*_i_*-responsive genes. In high P*_i_*, IP8 mediates the recruitment of SPX proteins to PHR transcription factors of the plant-specific MYB-CC class to repress PSI gene activation. When IP8 levels drop in low P*_i_* conditions, PHR–SPX complexes dissociate, leading to PSI gene activation and SPX protein degradation. Unlike the systemic P*_i_* response, local P*_i_* sensing of extracellular Pi availability is controlled by yet unknown processes (involving proteolysis), STOP1, a zinc finger transcription factor, which activates some of its target genes in low P*_i_*, such as ALMT1. For details, see the recent reviews (e.g., [80,87]). Reprinted from [16].

The mechanisms of P*_i_* sensing and response to its level in microalgae and yeast are also distinct: the transcription factor phosphate starvation response 1 (PSR1) is a key mediator of P starvation responses in algae, while PhoR/PhoB regulon is operating in bacteria (like *Escherichia coli)* and Pho80/Pho85/Pho4 regulon in yeast (*S. cerevisiae*), cf. panels Figure 5a–c.

PSR1 and other orthologs of PHR1/PHL, being master transcriptional regulators of the systemic P*_i_* starvation response, regulate the expression of many PSI genes in algae [85]. PSR1 belongs to a large Myb (Myeloblastosis) family of nuclear transcription factors common to eukaryotic organisms [70,86]. Overall, the discovery of SPX proteins and VTC homologs regulated via SPX domains across three phyla of microalgae might be indicative of a conserved mechanism of intracellular P*_i_* sensing via PP-InsP molecules [15,55,70].

Despite recent discoveries, the reports on the differential expression of VTC components and/or other polyP polymerases in green microalgae are still scarce in the literature. Current evidence shows that massive uptake of P*_i_* is followed by a transient spike of polyP biosynthesis, which is preceded by a peak of the expression of the gene encoding VTC in *Ch. reinhardtii* [32] and *Chlorella vulgaris* [39]. Similar indications of the relationships between VTC expression, polyP, and P availability were found in the coccolithophore *Emiliania huxleyi* [88,89] and the diatom *Thalassiosira pseudonana* [90,91].

Plausible mechanisms in control of the genes responsible for P*_i_* availability in bacteria (including cyanobacteria), yeast, and vascular plants are summarized in [16]; see also Figure 4. The latter is thought to be the closest to the one operation in eukaryotic microalgae, but the actual mechanism of P*_i_* sensing and gene expression control in these organisms can include a mix of components homologous to those in yeast and plants. It is also conceivable that a similar mechanism can be involved in the regulation of VTC-encoding genes. Briefly, under replete P*_i_* conditions, proteins with SPX domains bind 1,5-IP_8_ with high affinity (7–50 µM) [82]. The [SPX:1,5-IP_8_] complex then docks to the PHR1/PHL protein. As a result, its dimer dissociates, and the monomers interact with the MYB-like DNA-binding domain, blocking target gene activation [83]. Under low-P*_i_* conditions, a decrease in the 1,5-IP_8_ level declines the [SPX:1,5-IP_8_] complex formation and its interaction with other proteins promoting PHR1/PHL (hetero)oligomerization and activation of PSI genes via recognition of PHR1-binding sequence (P1BS) promoter elements. Positive and negative feedback loops for the expression of the genes harboring SPX domains are provided by the degradation of ligand-free SPX and P1BS-mediated protein activation, respectively. Obviously, in this regulatory framework, the expression of the VTC-encoding genes will be activated under replete P*_i_* conditions and repressed under a shortage of this nutrient.

## 6. Eco-Physiological Significance of Polyphosphate Polymerases

As outlined above, polyP polymerase is involved in the modulation of cellular P*_i_* storage by synthesizing polyP when P*_i_* is readily available in response to stimulation by high PP-InsPs levels. Its important role is participation in the responses of microalgae to fluctuations in the bioavailability of P [92]. Accordingly, the function of polyP polymerase is at the foundation of microalgal resilience to environmental stresses, especially nutrient shortage, and their ability to survive or even thrive in niches with a high level of competition, e.g., phytoplankton [93,94]. This might be an explanation of the famous “paradox of phytoplankton” (which is characterized by a large diversity of species, although a dominance of the most competitive is expected). This hypothesis also connects with the “starve out the competitor” strategy of competition in microbial communities [95]. Overall, microalgal species capable of rapid uptake and intracellular storing of P during short periods of its high availability will have the chance to outcompete other species less capable of doing so. All this highlights the importance of the intracellular P storing mechanisms and VTC as their important component for environmental fitness and competitiveness of microorganisms, including photosynthetic ones. In a similar way, cyanobacterial PPK1 is essential for the dynamic regulation of P metabolism, including hyperaccumulation of polyP, and to maintain physiological fitness under other nutrient (e.g., sulfur) starvation conditions [28]. Thus, knocking out of the *NoSPX2* gene, a VTC4 homolog from *Nannochloropsis oceanica*, diminished polyP levels and hindered the uptake of P*_i_*, confirming its role in polyP biosynthesis and P*_i_* homeostasis [63].

Another role of VTC, the long-term storage of energy, has been debated until now. On one hand, the P*_i_* units of polyP chains are connected by high-energy phosphor anhydride bonds, so the energy content of polyP is deemed to be significant [96,97]. Furthermore, knock-out strains lacking polyP polymerase display higher productivity [98]. On the other hand, the contribution of polyP synthesized by VTC to the total energy budget of the cell is questioned on the grounds of little or no effect of active polyP biosynthesis on carbon assimilation and biomass accumulation [15]. At the same time, the active biosynthesis of polyP normally takes place when the cell division rate is slow (during the lag phase after P*_i_* replenishment following P starvation or at the stationary phase), i.e., in situations when the energy demand is not so high as in the periods of active cell division. Therefore, the assessment of the real impact of polyP biosynthesis on assimilatory processes in the cell is complicated.

Although polyP is thought to function as a chaperone protecting protein against stress-induced misfolding [99], short-chain polyP can seemingly interfere with the folding of proteins in certain situations, e.g., when the P*_i_* is ample, but the energy is in short supply and cannot support the simultaneous elongation of many polyP chains [100,101] initiated outside the vesicles or vacuoles [43]. The symptoms of P*_i_* toxicity, namely cell division slowdown and cell structure disorganization, recently described in microalgae like *Chlorella* [100] and *Micractinium* [101], are hypothesized to be mediated by the ‘ectopic’ initiation of short-chain polyP [43] observed in P-starved microalgae upon abrupt replenishment of P*_i_*. This hypothesis explains the phenomenon of failed P*_i_* tolerance (which is normally quite high [50]) by analogy with the toxic effect of short-chain PolyP initially described in the yeast [44] and, recently, implied in *Chlorella regularis* [100,102]. The P toxicity syndrome links the failure of P*_i_* resilience of the microalgae initiation of polyP upon an abrupt rise in cytoplasmic P*_i_* at the VTC complexes, with subsequent shortage in ATP, the essential substrate for the polyP chain elongation, likely due to metabolic shutdown. It was shown (so far in yeast) that the cell can cope with the toxicity of non-vacuolar polyP by expressing polyphosphatases that degrade polyP [43]. Currently accumulated evidence suggests that the presence, activity of, and adequate energy supply to VTC are crucial determinants of the tolerance to the exogenic P*_i_* spikes. Namely, VTC mediates compartmentalization of the P*_i_* taken up in excess and translocation of the nascent polyP chain into the vacuole, thereby preventing the toxicity of short-chained polyP [50,101,103].

## 7. Implications for Biotechnology and Sustainability of P Usage

Currently, humanity relies very much on mined P minerals, which are finite, non-renewable, and non-uniformly distributed [104,105,106,107]. This problem is exacerbated by the extremely inefficient use of mined P and the products derived from it, especially mineral P fertilizers [105,108,109,110]. The usage of microalgal biotechnologies was proposed as a promising vehicle for making use of P resources more sustainably by means of capturing P from waste streams and re-routing it to agricultural ecosystems (see [111,112,113,114] and references therein).

As outlined above, the capability for the biosynthesis of polyP is important for the environmental fitness of microalgae. Therefore, it is no surprise that microalgae isolated from different habitats display the ability to synthesize polyP and that VTC4 is broadly conserved. Continuously facing fluctuating P availability, microalgae evolved to be very efficient at P acquisition and intracellular storage as polyP. This capability could be harnessed for sustainable usage of P, namely, to recycle P from waste streams and side streams and to mitigate water pollution. Indeed, the efficiency and competitiveness of the biotechnological solutions for sustainable use of P are determined by the rate and completeness of P bioremoval as well as by the size of the cell P quota (influenced profoundly by polyP accumulation capacity) of microalgal strains used in these solutions.

Environmental parameters, such as the N:P ratio, light intensity, or temperature influencing P accumulation in microalgal cells during wastewater treatment, are sensed and relayed in the cells to eventually modulate the expression of specific P-related genes and proteins (including VTC), but the underlying mechanisms of these processes are currently not known. The findings of [32,69] indicated that the differences in the expression of the genes participating in the biosynthesis of polyP cannot completely explain the variation in polyP yield and kinetics, even in the model microalga *Ch*. *reinhardtii*. Accordingly, the synthesis of polyP is believed to be regulated at the post-transcriptional level in microalgae, like in other organisms such as yeast, by interaction with PP-InP [8].

The considerations presented above make obvious the strong dependence of the capability of microalgal cultures to uptake and store P*_i_* in their cells and the performance of polyP polymerase [36,115]. Therefore, microalgal strains capable of rapid mobilization and uptake of P from diverse waste streams with subsequent conversion of the P*_i_* taken up into polyP are in high demand. These goals can be achieved by bioprospecting new strains with desired traits and/or by gaining better control of P metabolism. Another plausible way is cell selection, especially in the frame of an approach called “adaptive laboratory evolution” [116,117,118].

As the efficiency of microalgae in bioremoval of P is directly related to VTC, which is a very efficient enzyme [31], deeper insights into the structure, functioning, and regulation of VTC are needed to gain more control over P metabolism in microalgae. For example, an ambitious goal is constituted by the engineering of VTC for triggering polyP synthesis on demand, which can be indispensable for biotechnological P upcycling. Site-directed mutagenesis can be an approach to boosting the activity of VTC: for example, mutation of K24 and R31 amino acids to alanine in yeast VTC1 increased the activity of the whole VTC complex [30]. This might have direct consequences for the efficiency of microalgal technologies for the bioremoval of P from waste streams. It is also conceivable that microalgal strains with boosted VTC activity will not only be more efficient at P*_i_* scavenging but also less prone to short-chain polyP-mediated P*_i_* toxicity (see above) and hence more tolerant to P*_i_* spikes in the wastewater. Recently, polyP came into the spotlight as a valuable commodity [36,119], a potential antiviral [120] and anti-inflammatory agent [121], so boosting polyP accumulation in microalgae can be another driver for the engineering of VTC for productivity.

On the other hand, knocking down the genes responsible for the conversion of P*_i_* into polyP might be considered for enhancing the P deprivation effects, such as the stimulation of accumulation reserve lipids and other target compounds in microalgal cultures. Thus, disruption of the *Nospx2* gene encoding a putative polyP polymerase in *N. oceanica* considerably increased triacylglycerol and biomass accumulation under P*_i_* deficiency conditions [63]. A knock-out strain of the cyanobacterium *Synechocystis* sp. PCC 6803 lacking the polyphosphate kinase (*ppk*) and harboring a foreign ethylene-forming enzyme was obtained [98]. The engineered strain displayed higher ATP levels, faster growth, and better ethylene productivity than the wild-type under optimal conditions but lagged under stress [98]. So, the gain in productivity achieved by knocking out polyP-mediated biosynthesis of polyP comes at a cost of lower stress resilience, requiring more effort to maintain optimal cultivation conditions. Finally, a reservation should be made regarding the current legislation, which frequently prohibits the release of engineered strains into the environment, making their cultivation possible only in closed photobioreactors [122].

An adequate supply of energy is important for the uninterrupted flow of substrate (ATP) to VTC and hence for the unhindered accumulation of polyP. The preferred source of ATP for the biosynthesis of polyP is photophosphorylation, whereas dark respiration is less efficient in this regard; the formation of polyP is possible but is impaired profoundly under anaerobic conditions [123], likely due to the shortage of ATP. Therefore, the cultivation systems for the microalgal bioremoval of P*_i_* and the generation of polyP-rich biomass should be designed with the requirement of sufficient light energy supply in mind.

Finally, the representation and abundance of VTC in metagenomes can be a useful criterion in the selection of efficient algal–bacterial consortia for environmental biotechnology via functional profiling of the consortia’s metagenomes. Although many biotechnological applications of microalgae, such as the production of high-value metabolites, are based on axenic cultures, the use of the mutualistic consortia comprised by microalgae and associated heterotrophic bacteria quickly comes to the foreground, especially in nutrient bioremoval, bioremediation, and wastewater treatment [124]. This approach, using the mining of metagenomes to reveal the genetic determinants of the robustness and productivity of the consortia, quickly becomes a hot research direction and a basis for designing artificial consortia tailored to the application. Thus, consortia of “high-performance P absorbers” for application, e.g., in bioscrubbers used in wastewater polishing, can be engineered from individual strains featuring active VTC and hence a high capacity for taking up exogenic P*_i_* and storing it in the form of polyP. Although the mainstream method for metagenomic studies was high-throughput sequencing (HTS), the advent and rapid development of the third-generation nanopore sequencing made a recent breakthrough in this field. The development of novel bioinformatic algorithms for processing the metagenomic datasets opened new opportunities for the analysis of the structure and physiology of microalgal–bacterial communities. From the practical perspective, the new HTS techniques became time and labor savers in the discovery of new microalgae with a high potential for the accumulation of valuable metabolites, the biodegradation of hazardous micropollutants, and the biosequestration of nutrients from waste streams. The insights from both short-read and long-read metagenomics will form a solid foundation for the rational design of microalgal–bacterial consortia for biotechnology.

## 8. Conclusions and Outlook

Recent years have been hallmarked by important breakthroughs in our understanding of the genetic determinants, structure, and function of polyP polymerases in microalgae. Fundamental discoveries in the relevant model organism, *S. cerevisiae,* paved the way for these breakthroughs, which shed light on numerous aspects of P metabolism and storage in microalgal cells. These findings are important not only for basic science but for diverse applications in microalgal biotechnology as well. At the same time, many questions related to P metabolism in microalgae remain unanswered. Thus, the exact mechanism of polyP catabolism, especially of polyP compartmentalized in vacuoles and packed into large PolyP granules, is not known. In bacteria, polyP is degraded by exopolyphosphatase, which cleaves terminal phosphate off the polyP chains by hydrolysis or by endophosphatases, removing larger fragments of the polyP chain, and a similar mechanism operates in yeast. PolyP can also be catabolized via the reactions of phosphorylation of glucose, adenosine monophosphate, or NAD, involving polyP as P*_i_* donors [125]. However, as was inferred from the studies of *Ch. reinhardtii*, microalgae differ from yeast regarding polyP turnover since the microalga lacks homologs of the yeast exopolyphosphatase PPX1. Although Plouviez et al. [32] suggested that a NUDIX-box containing protein (see also [64,66]) may be responsible for polyP degradation in *C. reinhardtii*, microalgal pathways for the degradation of polyP remain vaguely known. Still, this question is of considerable importance, given that the existence of different polyP fractions in microalgal cells was discovered more than 60 years ago [126,127,128,129] but has not been fully explained yet. Whether different polyP polymerases are involved in the formation of low-mobility and high-mobility polyP fractions, or the main difference between these fractions is in their compartmentalization in the cell, remains unknown, too. At the same time, assessing the contributions of different polyP fractions to cell P quota and finding the conditions for the mobilization of low-mobility polyP fractions would be valuable insights for biotechnology. In this context, polyP toxicity and the role of polyP polymerases in its induction and mitigation also come into the foreground, but now our understanding of this problem is limited.

Of special importance are further structural studies of microalgal VTC-type polyP polymerases. One might expect that these studies will help to explain or even predict the species-specific differences in the capacity for PolyP accumulation and yield further mechanistic insights about post-translational regulation of polyP synthesis. Progress in this field requires establishing the protocols for the production of microalgal polyP polymerases by heterologous expression and subsequent in vitro characterization: Cliff et al. [15] rightly noted that current hypotheses about the regulation of PolyP synthesis in microalgae (e.g., by PP-InP binding to VTC) require experimental confirmation. At the same time, new and improved algorithms for modeling and prediction of VTC characteristics in silico (including the new machine-learning algorithms like AlphaFold 3 [130]) could be used as a screening tool for the assessment of the potential efficiency of PolyP synthesizing capacity in microalgae. This method will facilitate the selection of “top performing” microalgae in terms of P bioremoval from waste streams and accumulation of valuable PolyP. It is also an indispensable tool for the functional profiling of algal–bacterial consortia and the assessment of their potential for the same purpose. Both approaches will be useful for the development of microalgal biotechnologies for the sustainable usage of P.

Finally, the development of adaptive technologies for the upcycling of P from waste streams suitable for integration into conventional processes of waste treatment will be a major challenge in the near future. There is a basis to believe that deeper insights into the function and regulation of microalgal polyP polymerase function would help immensely to cope with this challenge.

## Figures and Tables

**Table 1:** The subunit composition of the VTC complex (according to the current understanding developed using yeast as a model organism [30]).

Subunit	Domains Harbored [PFAM Accession]	Function
Vtc1	Transmembrane domain, composed of three protomers (VTC1A, VTC1B, VTC1C)	Formation of the transmembrane channel guiding the nascent PolyP chain into the vacuole lumen
Vtc2		Auxiliary subunit; catalytically inactive ^1^
Vtc3	N-terminal SPX domain [PF03105]	Auxiliary subunit; not essential for VTC regulation ^1^
	TM1 domain	Discharging of polyP chain
Vtc4	Catalytic tunnel domain (triphosphate tunnel metalloenzyme, TTM domain) [PF09359]	Polymerization of polyP
	N-terminal SPX domain [PF03105]	Regulation (P*_i_* level sensing via PP-InsP)
	Transmembrane domain [PF02656]	Anchoring the whole complex to the vacuolar membrane and enabling translocation of the formed polyP chain into the vacuole
Vtc5	SPX domain [PF03105]	Accessory subunit for activation of the VTC complex. The only protein acting directly on the VTC complex to stimulate polyP production ^2^

^1^ homologous to VTC4. ^2^ presents only in yeast.

## Abbreviations

The following abbreviations are used in this manuscript:

P	Phosphorus
PHB	Poly-(R)-3-hydroxybutyrate
polyP	Polyphosphate
PP-InsP	Inositol pyrophosphate
PPK	Polyphosphate kinase
VTC	Vacuolar Transport Chaperone
